# Peroxisomal Membrane Contact Sites in Yeasts

**DOI:** 10.3389/fcell.2021.735031

**Published:** 2021-11-19

**Authors:** Amit S. Joshi

**Affiliations:** Department of Biochemistry and Cell and Molecular Biology, University of Tennessee, Knoxville, TN, United States

**Keywords:** membrane contact sites, peroxisomes, organelles, lipid droplet, mitochondria, endoplasmic reticulum

## Abstract

Peroxisomes are ubiquitous, single membrane-bound organelles that play a crucial role in lipid metabolism and human health. While peroxisome number is maintained by the division of existing peroxisomes, nascent peroxisomes can be generated from the endoplasmic reticulum (ER) membrane in yeasts. During formation and proliferation, peroxisomes maintain membrane contacts with the ER. In addition to the ER, contacts between peroxisomes and other organelles such as lipid droplets, mitochondria, vacuole, and plasma membrane have been reported. These membrane contact sites (MCS) are dynamic and important for cellular function. This review focuses on the recent developments in peroxisome biogenesis and the functional importance of peroxisomal MCS in yeasts.

## Introduction

Peroxisomes are conserved and highly dynamic organelles that are required for several metabolic processes, including beta-oxidation of fatty acids, reduction of reactive oxygen species, biosynthesis of plasmalogens and bile acids, oxidation of D-amino acids, and synthesis of precursors of cholesterol (Farré et al., 2019). In plants and yeasts, peroxisomes are the sites for the glyoxylate cycle (Kunze et al., 2006), whereas in some methylotrophic yeasts, peroxisomes are the sites for oxidation of methanol (Singh et al., 2020). Yeast is the favored model organism for investigating peroxisome formation and function. In *Saccharomyces cerevisiae*, *Yarrowia lipolytica*, and *Komagataella phaffii* (formerly known as *Pichia pastoris*), peroxisomes proliferate when grown in fatty acids such as oleic acid, whereas in *Ogataea polymorpha* (formerly known as *Hansunela polymorpha*) and *K. phaffii* peroxisomes proliferate when grown in methanol (Kunau and Hartig, 1992; Singh et al., 2020). Peroxisomes can alter their abundance based on external cues. Peroxisome number, size, and expression of enzymes required for metabolic pathways occurring in peroxisomes alter rapidly under peroxisome proliferating conditions (Hiltunen et al., 2003).

Cellular metabolic pathways are compartmentalized in different organelles. Byproducts of a pathway in one organelle might be utilized as a precursor in other organelles. Organelles dynamically interact with each other to facilitate efficient exchange of metabolites, organelle division, inheritance, signaling, and autophagy (Helle et al., 2013; Prinz et al., 2020). This interaction between organelles can occur at sites where membranes of two opposing organelles come in contact. These sites are often termed membrane contact sites (MCS) (Prinz et al., 2020). MCS are facilitated by one or more proteins that generate contact between two organelles and/or by protein–lipid interactions. These proteins that maintain MCS are known as tethers. There has been an exponential increase in the discovery of new tethers which has expanded our understanding of MCS and organelle dynamics. Similar to other organelles, peroxisomes have several MCS that enable dynamic interaction with various organelles (Shai et al., 2016; Sargsyan and Thoms, 2020; Silva et al., 2020). This review will focus on recent advances in peroxisome biogenesis, addressing the importance of MCS in the formation and function of peroxisomes in yeasts.

## Peroxisome–ER Contact Sites

**FIGURE 1:**
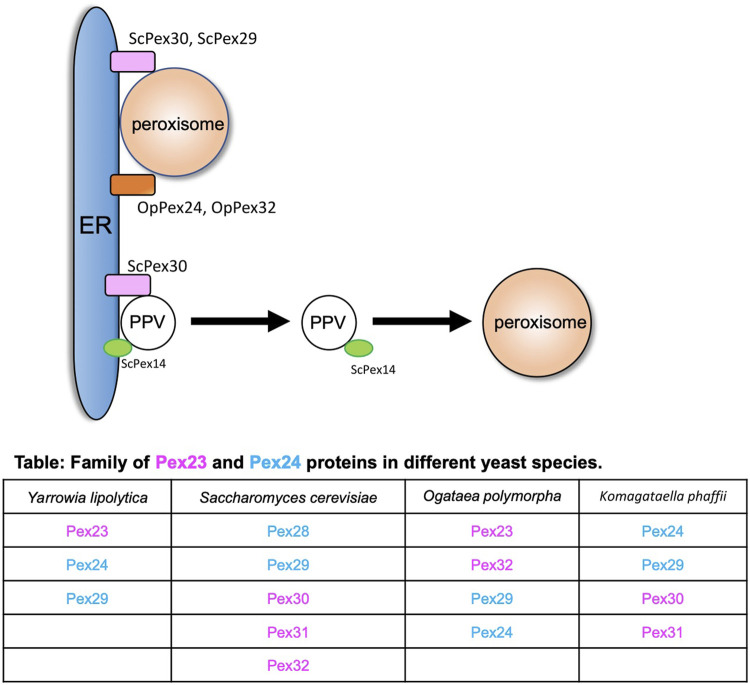
Peroxisome–ER contact sites. Peroxisome and PPVs contact the ER at subdomains enriched with the Pex23 and Pex24 protein families. ScPex30 and ScPex29 and OpPex24 and OpPex32 are at the ER–peroxisome contact sites. ScPex30 is at ER subdomains where ScPex14-containing nascent PPVs are formed. The table contains the family of Pex23 (magenta) and Pex24 (blue) proteins in different yeasts.

The endoplasmic reticulum (ER) is the major site for the synthesis of cellular proteins and lipids (Joshi et al., 2017). In yeasts, peroxisomes mainly follow the growth and division model where a new peroxisome is formed from the preexisting one (Goldman and Blobel, 1978; Lazarow, 1983). As peroxisomes grow, they need lipids and proteins, and they acquire these from the ER. In the early electron micrographs, peroxisomes were found to be in close proximity with the ER (Baudhuin et al., 1965; Tsukada et al., 1968; Novikoff and Novikoff, 1972). Peroxisomes can receive membrane lipids either via vesicles that fuse with the existing peroxisomes or by non-vesicular lipid transport that occurs between the ER and peroxisomes (Raychaudhuri and Prinz, 2008). The peroxisomal membrane proteins (PMPs) are also targeted to the peroxisomes via the ER (Geuze et al., 2003; Perry et al., 2009; Titorenko and Rachubinski, 2009; Agrawal and Subramani, 2013; Tabak et al., 2013; van der Zand et al., 2010; Kim and Hettema, 2015). Alternatively, PMPs can be transported to existing peroxisomes through pre-peroxisomal vesicles (PPVs), also known as ghost vesicles (Agrawal et al., 2016; Joshi et al., 2016). PMPs can also sort directly to the membranes of peroxisomes (Mayerhofer, 2016). The homologs of the Pex23 and Pex24 family of peroxins in various yeasts (Figure 1) that reside in the ER membrane play an important role at peroxisome–ER contact sites (Yan et al., 2008; David et al., 2013; Mast et al., 2016; Wu et al., 2020; Ferreira and Carvalho, 2021; Jansen et al., 2021). In *S. cerevisiae*, ScPex29 and ScPex30, whereas in *O. polymorpha*, OpPex24 and OpPex32 accumulate at the peroxisome-ER MCS (David et al., 2013; Mast et al., 2016) (Figure 1). ScPex30 and ScPex31 have a reticulon homology domain (RHD) and tubulate the ER membrane. Unlike the reticulon proteins, ScPex30 and ScPex30 are located in discrete regions in the ER subdomains (Joshi et al., 2016; Ferreira and Carvalho, 2021). A proteomic screen was performed to identify the proteins that interact with ScPex30, which revealed that ScPex30 forms a complex with the ER-localized reticulon and reticulon-like proteins, Rtn1, Rtn2, and Yop1, at peroxisome–ER contact sites (David et al., 2013; Mast et al., 2016). Loss of ScPex30 leads to an increase in the mobility of peroxisomes, suggesting that ScPex30 could act as a possible peroxisome–ER tether. The absence of either ScPex30 or reticulon proteins leads to an increase in peroxisome biogenesis (David et al., 2013). However, other reports mention that deletion of Pex30 in *S. cerevisiae* and *K. phaffii* leads to a decrease in peroxisome number and the rate of peroxisome formation (Yan et al., 2008; Joshi et al., 2016). Deletion of OpPex24 and OpPex32 leads to defects in peroxisomal matrix protein import, membrane growth, and peroxisome proliferation, possibly due to defects in peroxisome–ER contact. Using an artificial peroxisome–ER tether, the defects observed in cells devoid of OpPex24 and OpPex32 were restored. Additionally, OpPex11 is required for Pex32-dependent peroxisome–ER contact (Wu et al., 2020). The Pex23 and Pex24 protein families have an uncharacterized dysferlin domain (Yan et al., 2008), which is predicted to bind other proteins. Is it possible that the dysferlin domain might recruit additional proteins to form a complex at the contact sites? It is important to identify the proteins and lipids enriched at these ER subdomains to understand the molecular mechanisms of peroxisome growth at peroxisome–ER contact sites.

In addition to the growth and division model, peroxisomes also follow the *de novo* biogenesis model where new peroxisomes can be generated in the cells from existing donor membranes (Hoepfner et al., 2005). In yeasts, new peroxisomes form from the ER membrane, whereas in mammalian cells they also form from the mitochondrial outer membrane (Hoepfner et al., 2005; Kim et al., 2006; Sugiura et al., 2017). The *de novo* peroxisome biogenesis begins with the formation of PPVs in the ER membrane. Once formed, PPVs acquire PMPs that enable the import of matrix proteins (Knoops et al., 2014). How the PMPs are targeted to the PPVs is not known. In yeasts, Pex3 and Pex19 are required for the early steps of PPV formation as cells devoid of Pex3 or Pex19 do not contain any PPVs (Agrawal et al., 2011; Hettema et al., 2000; Lam et al., 2011; Tam et al., 2005). However, it was recently shown that cells devoid of Pex3 and Pex19 contained PPVs. In fact, these PPVs contain several PMPs such as Pex14, Pex13, and peroxisomal matrix proteins (Knoops et al., 2014; Wróblewska et al., 2017). Thus, we still do not understand how PPVs form in the ER membrane in yeast cells. ScPex30 localizes to ER subdomains where ScPex14-containing new PPVs form. Newly synthesized ScPex14 colocalizes with ScPex30 subdomains and is possibly transported into new PPVs (Figure 1) (Joshi et al., 2016). Even though ScPex30 and ScPex31 are not essential for PPV formation, loss of ScPex30 and ScPex31 leads to less mobile and highly clustered PPVs wrapped with the ER membrane (Joshi et al., 2016). Consistent with this, cells devoid of Pex30 and Pex31 also exhibit defects in peroxisome morphology and abundance. Other PPVs contain PMPs such as Pex2, Pex10, and Pex12 that require Pex3 and Pex19 for their formation (Agrawal et al., 2016). Whether these PPVs also form at Pex30 subdomains is not known.

The majority of Pex23- and Pex24-like proteins in different yeasts are localized at ER subdomains (Yan et al., 2008; Joshi et al., 2016, 2018; Mast et al., 2016; Wang et al., 2018; Wu et al., 2020; Ferreira and Carvalho, 2021). These ER subdomains are either sites of organelle formation or contact sites for other organelles such as peroxisomes and lipid droplets (LDs). How these proteins act as tethers is not known. We also do not understand the segregation of discrete ER subdomains, sorting of PMP proteins to PPV budding sites, and the PPV budding machinery.

## Peroxisome–LD Contact Sites

**FIGURE 2:**
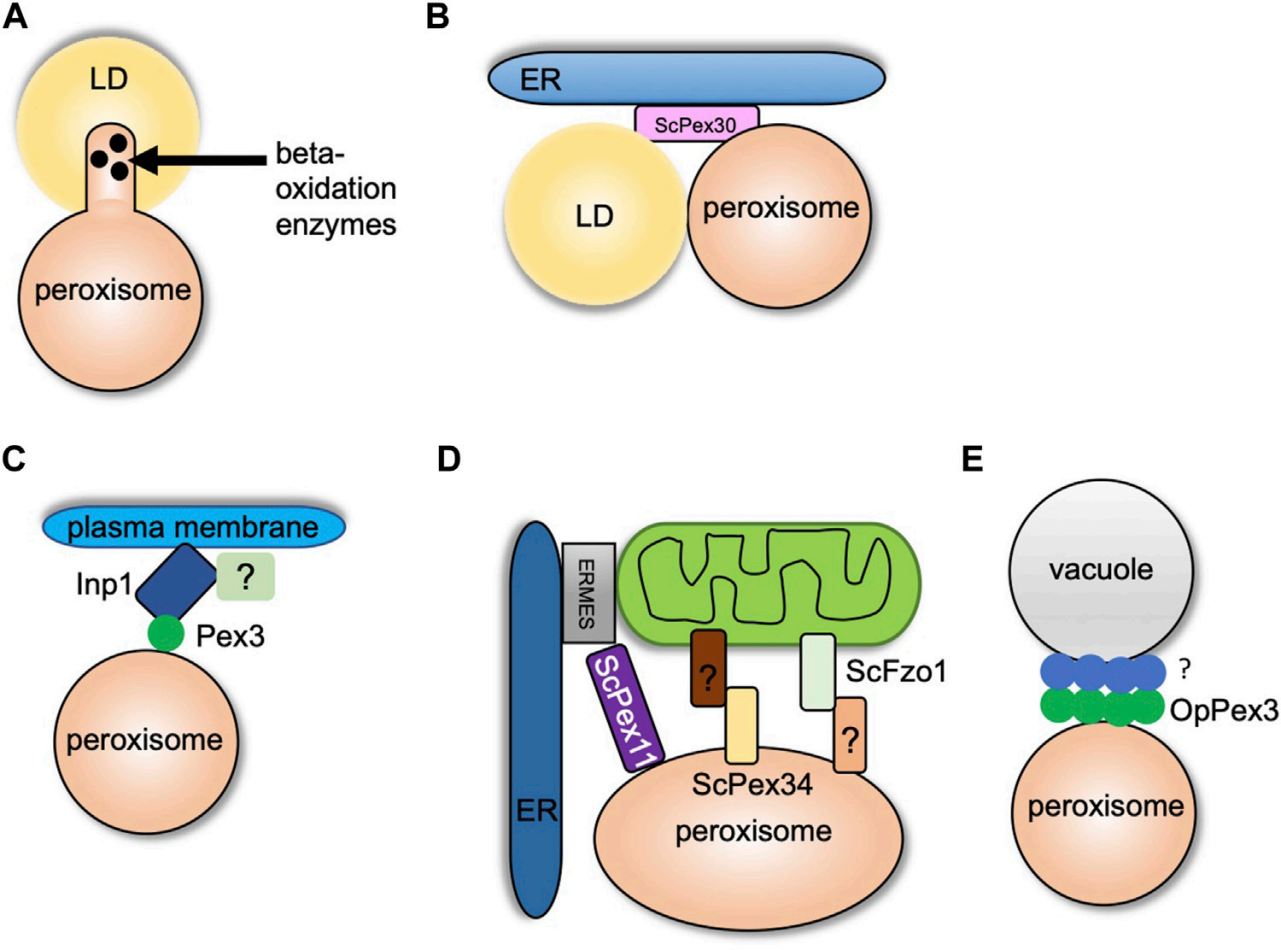
Peroxisome contact sites in yeasts. **(A)** and **(B)** indicate peroxisome–LD contact sites. **(A)** Formation of pexopodia enriched in beta-oxidation enzymes into the core of LDs in peroxisome-proliferating growth conditions. **(B)** LDs and peroxisomes interact at the ScPex30 ER subdomains. **(C)** Peroxisome–plasma membrane contact site. Inp1 binds to the plasma membrane at sites enriched in PI (4,5) P2 with another possible binding partner. Inp1 tethers peroxisome to the plasma membrane by binding Pex3 localized on the peroxisomal membrane. **(D)** Peroxisome–mitochondria contact sites. Pex11 binds Mdm34 of the ERMES complex at the ER–mitochondria contact sites. Fzo1 on mitochondrial membrane contacts an unknown binding partner on peroxisomes, and Pex34 on peroxisomal membrane binds an unknown binding partner on the mitochondria. **(E)** Peroxisome–vacuole contact sites. OpPex3 accumulates at the peroxisome–vacuole contact sites in peroxisome-proliferating conditions or when overexpressed. The OpPex3 binding partner on the vacuolar membrane is unknown.

While peroxisomes degrade lipids, LDs are known to store neutral lipids such as triacylglycerol (TG) and sterol esters (SE). LDs are storage organelles found in most eukaryotic cells (Olzmann and Carvalho, 2019). Both peroxisomes and LDs are similar in function as they are mainly involved in maintaining lipid homeostasis. However, structurally these organelles are very different (Joshi and Cohen, 2019). LDs are unique as they have a core that mainly consists of neutral lipids that are surrounded by a phospholipid monolayer (Cohen, 2018). LDs interact with several organelles such as the ER, mitochondria, vacuole/lysosome, and peroxisomes. Here we focus on the crosstalk between LDs and peroxisomes. Several reports provide strong evidence of peroxisome–LD contact (Novikoff et al., 1980; Schrader, 2001). In *S. cerevisiae* cells, peroxisomes form finger-like membrane extensions called pexopodia into the core of LDs when grown in oleic acid (Figure 2A). The electron micrographs show the fusion of a single leaflet of LDs with the outer leaflet of the peroxisomal membrane which enables direct contact of the inner leaflet with the core of the LDs. The peroxisomal membrane extensions were enriched in fatty acid oxidation enzymes, suggesting lipid breakdown at these sites (Figure 2A). In cells devoid of functional peroxisomes such as *pex5* mutants, several membrane extensions, termed as gnarls, in and out of the LDs were observed. These membranes were devoid of any fatty oxidation enzymes (Binns et al., 2006). When yeast cells are grown in fatty acid–enriched growth medium, several other organelles such as the ER and mitochondria are tightly associated with peroxisomes and LDs (Sargsyan and Thoms, 2020). As mentioned in the section *Peroxisome–ER Contact Sites*, ScPex30 is an ER-resident protein localized at ER subdomains where PPVs, as well as peroxisomes, contact the ER. Interestingly, new LDs also form at ScPex30 ER subdomains. Using three-color live cell imaging, it was discovered that PPVs and peroxisomes associate with LDs at ScPex30 subdomains in the ER membrane (Joshi et al., 2018) (Figure 2B). Even though peroxisomes and LDs have been shown to associate, a physical tether has not yet been identified in yeasts. However, in mammalian cells, M1 spastin, a membrane-bound AAA ATPase, on the LDs physically interacts with ABCD1 on the peroxisomal membrane to facilitate fatty acid trafficking from LDs to peroxisomes by recruiting ESCRTIII proteins to the LDs (Chang et al., 2019).

Several proteins required for peroxisome and LD biogenesis are also shared. Peroxisome–LD contacts increase in fasting conditions where LDs undergo lipolysis by adipose triglyceride lipase (ATGL). Pex5 stimulates the translocation of ATGL on the LD surface, possibly at peroxisome–LD contact sites (Kong et al., 2020). In another study, it was demonstrated that farnesylated Pex19 is essential for targeting of UBDX8, an LD protein, to Pex3-enriched ER subdomains (Schrul and Kopito, 2016). The peroxisomal protein fatty acyl CoA reductase 1 (Far1) is targeted to LDs upon increased triglyceride synthesis. Far1 exhibits dual topologies in peroxisomes and LDs dependent on cellular lipid metabolism (Exner et al., 2019). It is clear that there is a crosstalk among the ER, peroxisomes, and LDs in yeast and mammalian cells (Joshi et al., 2018). The interplay may facilitate dual localization of proteins and movement of lipids between ER, LDs, and peroxisomes and allows cells to cope with the metabolic requirements.

## Peroxisome–Plasma Membrane Contact Sites

The peroxisome population is maintained by regulating transport and inheritance into the daughter cells (Knoblach and Rachubinski, 2016). In yeasts, Inp1 and Inp2 are the peroxisome inheritance proteins that control the retention and inheritance of peroxisomes, respectively. In the absence of Inp1, mother cells are unable to retain peroxisomes, and Inp2 is a peroxisomal membrane protein that interacts with Myo2 and is essential for peroxisome inheritance in daughter cells (Fagarasanu et al., 2005; Knoblach and Rachubinski, 2019; Saraya et al., 2010). In yeasts, it was suggested that Inp1 acts as a “molecular hinge” by binding to Pex3, which is present on both the ER and peroxisome membrane with its N and C termini (Knoblach et al., 2013). The peroxisome–ER contact site generated by Inp1 and Pex3 is required to retain peroxisomes in the mother cell. Two recent studies contradicted the “molecular hinge” model as the new model showed that Inp1 is a component of the tether at the peroxisome–plasma membrane contact site (Hulmes et al., 2020; Krikken et al., 2020). The N-terminal 100 amino acids of ScInp1 localizes to the plasma membrane possibly by binding to PI-(4,5)-P2. The N-terminal fragment (minimal tether) of ScInp1 is enough to retain peroxisomes at the cell periphery by tethering them to the plasma membrane. Importantly, the full-length ScInp1 and the ScInp1 minimal tether localized to the cell periphery spatially resolved from the ER on the peroxisomal foci in close proximity to the plasma membrane. The ScInp1 minimal tether was not able to position peroxisomes, causing clustering of peroxisomes at the bud neck. Thus, the full-length ScInp1 might have additional contacts for proper positioning of the peroxisomes, which could be mediated by unidentified factors at the cell cortex or by other organelles such as the cortical ER. ScPex30 was found to interact with ScInp1. However, the loss of ScPex30 does not have any effect on the retention of peroxisomes. If the ER plays a role along with Inp1 in peroxisome retention, there might be proteins similar to ScPex30 that are required for the correct positioning of peroxisomes (Hulmes et al., 2020) (Figure 2C). Interestingly, another study also showed that OpInp1 is required for the formation of peroxisome–plasma membrane contact and peroxisome retention. As mentioned in the section *Peroxisome–ER Contact Sites*, OpPex32 and OpPex24 are required for peroxisome–ER contact. Cells devoid of OpInp1 and OpPex32 did not have an additive defect in peroxisome retention (Krikken et al., 2020). These findings in *S. cerevisiae* and *O. polymorpha* demonstrate that Inp1 directly associates with the plasma membrane and binds to Pex3 on the peroxisomal membrane to form peroxisome–plasma membrane contact. Additional components required for peroxisome tethering to the plasma membrane, peroxisome positioning, and peroxisome retention remain to be investigated.

## Peroxisome–Mitochondria Contact Sites

Peroxisomes and mitochondria have a tight metabolic interaction, especially in peroxisome-inducing growth conditions. Both the organelles also share fission protein machinery that regulates the abundance and function of these organelles (Ast et al., 2013). In *S. cerevisiae*, the enzymes for beta-oxidation of fatty acids are localized in peroxisomes. However, the byproduct of this pathway, acetyl-CoA, is transported to the mitochondria where it is utilized by the tricarboxylic acid cycle (TCA). It was shown that peroxisomes are juxtaposed at the mitochondria subdomains in contact with the ER and sites enriched in the pyruvate dehydrogenase (PDH) complex (Cohen et al., 2014). The PDH complex dehydrogenates pyruvate to acetyl-CoA, which is utilized in the TCA cycle (Kresze and Ronft, 1981). Another report demonstrated that ScPex11, a peroxisome membrane protein, interacted with ScMdm34, a component of the ER–mitochondria tether, ERMES, and thus could serve as a peroxisome–mitochondria tether (Mattiazzi Ušaj et al., 2015) (Figure 2D). Peroxisomes are also juxtaposed at ER–mitochondria contact sites during pexophagy. Decreased association of ScMdm34 and ScPex11 leads to defects in pexophagy (Liu et al., 2019). Using a split fluorescence reporter, an elegant high-content microscopy screen demonstrated that ScPex34 and ScFzo1, homolog of mitofusin 1 and 2, could tether peroxisomes and mitochondria (Figure 2D). ScFzo1 oligomerization is essential for mitochondrial fusion (Griffin and Chan, 2006). When overexpressed, ScFzol increases the peroxisome–mitochondria contact. A fraction of ScFzo1 was also found on the peroxisomes, suggesting a homotypic interaction between mitochondrial and peroxisomal ScFzo1. However, endogenously expressed ScFzo1 was not found on the peroxisomal membrane, hinting at other binding partners present on peroxisomes. Overexpression of ScPex34 additionally expanded the peroxisome–mitochondria contact even in the absence of ScFzo1. ScPex34 but not ScFzo1 is required for the transport of byproducts of the beta-oxidation pathway such as citrate and acetyl-CoA from peroxisomes to the mitochondria (Shai et al., 2018). ScPex34 and ScFzo1 are not part of the same tethering complex. The ScPex34 binding partner on the mitochondrial membrane remains to be investigated.

## Peroxisome–Vacuole Contact Sites

Previously, peroxisome–vacuole contact has been observed during micropexophagy in *O. polymorpha* (Bellu et al., 2001). In nitrogen starvation conditions, the vacuole membrane wraps around the peroxisome to be degraded. In the recent study, extensive peroxisome–vacuole contact was reported in cells grown in peroxisome-inducing conditions. The *O. polymorpha* cells were grown in methanol to induce peroxisomes. OpPex3 accumulated at the peroxisome–vacuole contact sites. When cells were grown in glucose, a condition known to repress peroxisome proliferation, endogenous OpPex3 was not observed at the peroxisome–vacuole contact sites. However, when OpPex3 was overexpressed, it accumulated at the peroxisome–vacuole contact sites. The proposed model is that OpPex3 acts as an anchor protein on the peroxisomal membrane at the peroxisome–vacuole contact sites like the peroxisome–plasma membrane contact sites where it binds to OpInp1 (Wu et al., 2019) (Figure 2E). The binding partner for OpPex3 on the vacuolar membrane is not known. What is the function of the peroxisome–vacuole contact site? It is speculated that the peroxisome–vacuole contact sites could be important for membrane lipid transport from the vacuole to the peroxisome, which is essential for the growth of peroxisomes. How does Pex3 enhance lipid transport at these contact sites? Overexpression of OpPex3 in glucose-grown cells increases peroxisome–vacuole contact. Does this lead to an increase in peroxisome size? The mechanism and the function of peroxisome–vacuole contact sites need to be determined.

## Concluding Remarks

There are many outstanding questions about the formation, function, and regulation of peroxisomal membrane contacts. New tools to study MCS, including correlative light and electron microscopy, electron tomography, multispectral imaging, and super-resolution microscopy, have provided crucial information that multiple organelles interact with each other to respond to cellular needs and environmental cues. Unraveling new MCS and tethers will integrate cellular metabolism, signaling, and organelle dynamics at MCS. Elegant microscopy screens (Kakimoto et al., 2018; Shai et al., 2018) coupled with the awesome power of yeast genetics will provide a unique opportunity to discover novel peroxisomal MCS. It is evident that more than two organelles can be involved at MCS. Also, more than one contact can be established independently between two organelles. Future investigations must take an inclusive approach by focusing on the dynamics of multiple organelle interactions. The role of peroxisomes in cellular homeostasis and their association with severe neurological disorders, including Zellweger syndrome, X-linked adrenoleukodystrophy, and aging, emphasize the importance of the detailed study of the molecular mechanisms of new and existing peroxisomal MCS.
